# Toward a Personalized Basal Tuner for Detecting Basal Rate Inaccuracies in Type 1 Diabetes Mellitus Without Meal Data: Algorithm Development and Retrospective Validation Study

**DOI:** 10.2196/72769

**Published:** 2025-11-26

**Authors:** Daniel Gasca Garcia, Hood Thabit, Paul W Nutter, Simon Harper

**Affiliations:** 1 Department of Computer Science, University of Manchester, Kilburn Building, Oxford Rd, Manchester, M13 9PL, United Kingdom, 44 7565135239; 2 Division of Diabetes, Endocrinology and Gastroenterology, Faculty of Biology, Medicine and Health, University of Manchester, Manchester, United Kingdom; 3 Diabetes, Endocrine & Metabolism Centre, Manchester Royal Infirmary, Manchester University NHS Foundation Trust, Manchester, United Kingdom

**Keywords:** personalized basal tuner, PBT, meal detection, adaptive basal insulin, blood glucose management, type 1 diabetes mellitus, T1DM

## Abstract

**Background:**

Basal rate (BR) adjustment is crucial for managing type 1 diabetes mellitus, accounting for 30% to 50% of total daily insulin needs. All current closed-loop systems revert to the user’s usual pump BR (known as manual mode) in the event of closed loop failure. Furthermore, access to closed-loop systems remains relatively low in low- and middle-income countries and among those without suitable health insurance. Accurately adjusting the BR remains challenging, leading to hypo- or hyperglycemia, and research on optimizing the BR is limited.

**Objective:**

This study proposed an adaptive algorithm that uses continuous glucose monitoring data to identify BR inaccuracies without requiring meal intake information.

**Methods:**

The OhioT1DM dataset formed the basis for implementing this methodology. Each composite day was generated by excluding bolus insulin profiles lacking meal intake information and by calculating hourly blood glucose (BG) relative levels along with their corresponding reliability measures, enabling assessment of deviations from the recommended BR (ie, a BG relative change of 0 mg/dL). Both a noninferiority analysis and a classification precision metric were used to assess the practicality of this approach compared to using meal data.

**Results:**

Data from 12 participants showed noninferiority of the no-meal method: using a 20% noninferiority margin on absolute BG relative change, 9 of 12 participants met the criterion (1-sided *P*<.05). Classification precision was 73.9% (139/188) of meals correctly classified on average per participant (SD 11.8%; 95% CI 67.2%-79.7%). The daily cumulative BG average was 200.6 mg/dL (SD 61.7 mg/dL; 11.1 mmol/L, SD 3.4 mmol/L; 95% CI 161.4–239.8 mg/dL), with peak values reaching 270.15 mg/dL (14.99 mmol/L). Furthermore, 99.3% (286/288) of the BG relative values (SD 0.5%; 95% CI 97.5%‐99.8%) that were unaffected by external factors were associated with incorrect BR settings, with deviations ranging from −25.5 to 46 mg/dL (−1.58 to 2.59 mmol/L).

**Conclusions:**

Current strategies to optimize BR settings are inadequate, and our approach of a personalized basal tuner (PBT) helps better analyze BR without relying on meal intake information. Indeed, without an optimally set BR, in the event of the closed loop reverting to manual mode, patients may be exposed to persistent hypo- or hyperglycemia, leading to safety and efficacy issues. Future work will focus on generating BR recommendations through the application of this algorithm in clinical practice to assist clinicians in setting BR in low- and middle-income countries, where closed-loop systems are not prevalent, to help increase time in range.

## Introduction

### Background

The current state of the art in insulin delivery is automatic insulin delivery, also known as closed-loop systems [1]. In real-world settings—such as with the open source loop system—these have been shown to improve both glycemic control and quality of life [2]. However, all existing closed-loop systems revert to the user’s preset pump basal rate (BR) manual mode in the event of system failure. Specific closed-loop systems also use the pump BR for other reasons, such as serving as an integral part in the control algorithm functionality (Tandem t:slim) [3] or as a safety backup (reverting to manual mode) if the user has been hyperglycemic for a set time (Omnipod 5) [4].

While high-income countries are transitioning to closed-loop systems, those in low- and middle-income countries (LMICs) and without suitable health insurance have limited access to closed-loop systems [5,6]. Therefore, without an optimally set BR, a closed-loop system reverting to manual mode may expose patients to persistent hypo- or hyperglycemia, compromising both safety and efficacy. This is particularly crucial as basal insulin contributes approximately 30% to 50% of total daily insulin use in individuals with type 1 diabetes mellitus (T1DM) [7,8].

Recent evidence indicates that insulin requirements, including BR, do not always follow predictable circadian or clinically assumed patterns, with unexpected deviations occurring as frequently as expected ones [9]. Such variability underscores the need for regular assessment and adjustment of BR settings to ensure stability across different operational modes and clinical contexts. Accordingly, the proposed algorithm is clinically relevant in several scenarios: (1) when a closed-loop system fails and reverts to manual mode; (2) in devices that incorporate fixed basal profiles as part of their control strategy (eg, the Tandem t:slim); (3) when safety protocols trigger a manual mode reversion after sustained hyperglycemia (eg, the Omnipod 5); and (4) in settings where closed-loop technology is unavailable due to cost, insurance coverage, or regulatory constraints, such as in many LMICs. In all these cases, an accurately set BR remains essential for maintaining glycemic stability.

### Challenges in BR Optimization

Traditionally, establishing BR has involved a series of fasting tests, each lasting 6 to 12 hours and conducted at different times of the day. The results are used to fine-tune, retest, and record BR settings. While effective, this method can be challenging for both patients and clinicians [10] and is less commonly used in routine practice, where adjustments are often made based on continuous glucose monitoring (CGM) or finger-prick blood glucose (BG) values. Once the initial BR is determined, the clinician’s recommendations remain unchanged for several months between clinic visits. This approach is particularly concerning for children and adolescents, whose physiological needs evolve rapidly, making it difficult to ensure that their requirements are adequately adjusted and addressed [11-14].

### Objective

The objective of this study was to evaluate whether accurate assessment of basal insulin rates can be achieved without requiring meal information. Using 45 days of CGM and basal insulin delivery data, the algorithm generates a composite 24-hour basal profile designed to support stable and safe glycemic control, particularly in scenarios in which a closed-loop system fails and reverts to manual mode. Current guidelines recommend a time in range (TIR) of at least 16.8 hours per day (70%), corresponding to BG levels between 70 and 180 mg/dL (3.9‐10 mmol/L) for that proportion of time [15,16]. While this work focused on demonstrating the nonnecessity of meal data for such assessments, the application of these results to guide BR adjustments will be addressed in future research.

### Related Work

Several studies have explored strategies to set up basal insulin delivery for improved glycemic control in individuals with T1DM. Run-to-run adaptive control strategies using model predictive control dynamically adjust basal insulin based on daily glycemic patterns to enhance glucose stability and reduce hypoglycemia risk [17]. Similarly, iterative learning control has been applied to optimize basal insulin in multiple daily injection therapy, leveraging historical BG data to personalize and refine dosing over time [18]. Additionally, a multivariate learning framework has been proposed for artificial pancreas systems, allowing for continuous adaptation of basal insulin rates in response to individual variability and long-term changes in glycemic behavior [19]. Finally, a multiagent reinforcement learning method has been used to adjust both basal and bolus insulin dosing [20].

### Study Contribution

Unlike methods that depend on complex models and real-time adjustments, the personalized basal tuner (PBT) algorithm provides a simpler, more practical solution for evaluating BR without the need for meal data. Additionally, updating calculations on a 45-day basis may improve the performance of certain closed-loop systems that incorporate BR as a key component of their algorithms. This approach could also enhance the safety of these systems when reverting to manual mode. Furthermore, it supports clinicians in LMICs, where closed-loop systems are less common, by aiding in BR adjustments to improve TIR and better manage BG fluctuations unrelated to meal intake.

### Aim and Hypothesis

The primary aim of this study was to develop and evaluate an algorithm capable of identifying and removing BG excursions caused by mealtime insulin boluses without requiring meal information. The hypothesis was that this approach would be noninferior and reasonably precise compared with methods using meal data, thereby supporting its use in scenarios in which meal information is unavailable or unreliable.

## Methods

### Ethical Considerations

This study used the OhioT1DM dataset [21], originally collected under Institutional Review Board approval at Ohio University (National Institutes of Health grant 1R21EB022356). Access to the dataset requires a data use agreement signed between Ohio University and the requesting institution, as stated in the dataset descriptor. In this work, access was provided to the research team at the University of Manchester under such an agreement, and all records were fully deidentified before release. As this was a secondary analysis of anonymized data, no additional ethics approval was required locally in accordance with University of Manchester policy on the use of anonymized secondary data.

### Participants

This study used data from 12 adults with T1DM included in the OhioT1DM dataset [21]. For each participant, approximately 45 consecutive days of CGM, insulin delivery, and self-reported life event information were available. Participant demographics are summarized in Table 1. Although the cohort was modest in size, each record provided a rich multimodal profile that included CGM, insulin therapy, and contextual events. This level of longitudinal detail supports reproducible analyses and methodological development despite the limited number of participants.

**Table 1.** Demographic characteristics of participants in the OhioT1DM dataset relevant to the personalized basal tuner algorithm.

ID	Sex	Age group (y)	Pump model	Cohort year^a^	Activity reported^b^
540	Male	20‐40	630G	2020	No
544	Male	40‐60	530G	2020	No
552	Male	20‐40	530G	2020	No
567	Female	20‐40	630G	2020	No
584	Male	40‐60	530G	2020	No
596	Male	40‐80	530G	2020	No
559	Female	40‐60	530G	2018	Yes
563	Male	40‐60	530G	2018	Yes
570	Male	40‐60	530G	2018	Yes
575	Female	40‐60	530G	2018	Yes
588	Female	40‐60	530G	2018	Yes
591	Female	40‐60	530G	2018	Yes

a The dataset includes patients from different cohorts and with different pump models.

b “Activity reported” indicates whether the participant provided physical activity data.

### Materials

Each participant’s dataset includes 20 distinct variables viewable through the open-source OhioT1DM Viewer (Ohio University) [21]. For the scope of this study, the following specific variables were extracted and analyzed for each individual: BG, BR, bolus insulin doses, meal data, and baseline step count.

### Main Algorithm

**Figure 1.**
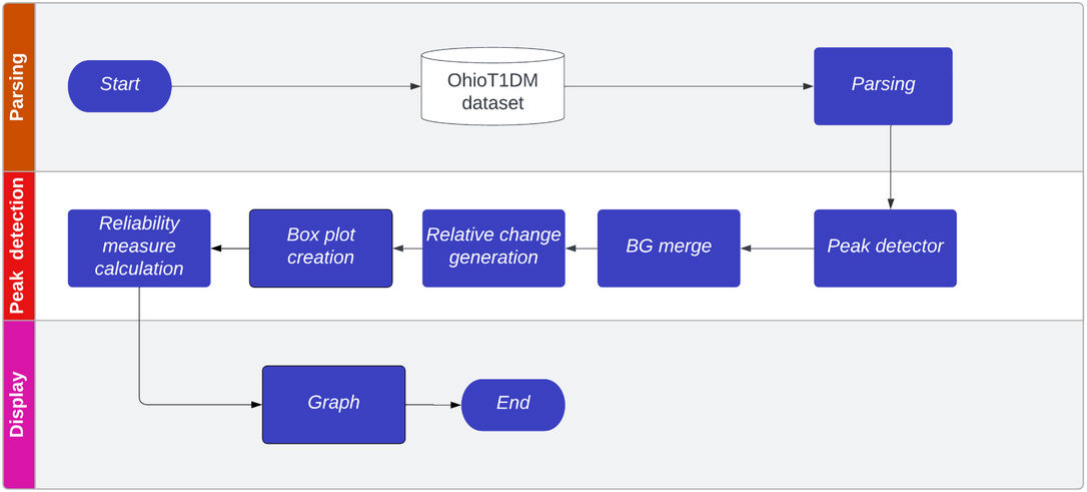
The developed algorithm is organized into 3 stages: one focused on preprocessing blood glucose (BG) data, another focused on peak detection and evaluation of basal rate (BR), and the final stage focused on display and analysis. The main objective was to highlight and evaluate aspects related to BR.

The current algorithm functions as a sequential pipeline designed for the analysis of T1DM data, with distinct stages designed to identify inaccuracies in historical BR (Figure 1).

The first stage involves parsing CGM and insulin delivery data, which are downloaded from the corresponding devices. The second stage applies a peak detection algorithm to filter out BG readings influenced by meal intake, preserving readings from fasting periods as much as possible. The primary goal is to remove meal-related peaks even when the meals are not recorded. This process is applied to all historical data, and relative changes in BG are then calculated on an hourly basis to quantify deviations from the optimal target of 0 mg/dL, which serves as the conceptual baseline. In clinical practice, once the clinician determines the optimal basal insulin dose, it is expected that fasting glucose will remain stable or show minimal deviation. Afterward, box plots are generated to analyze and visualize the distribution of BG relative changes. Finally, a metric based on the number of data points is calculated to assess the reliability of the obtained values. The final stage focuses on displaying results and evaluating the findings (see the Results and Discussion sections for further reference).

In summary, this variant of the algorithm takes a different approach by avoiding the necessity of meal information. Instead, it leverages historical data to characterize BG peaks, effectively removing them and, thus, providing clear BG readings unaffected by external factors such as meals or mealtime insulin bolus delivery. This method offers an approach to evaluating BR in historical BG readings adaptable to any patient.

### Peak Detection Analysis

Peak detection serves the purpose of identifying and removing the influence of mealtime insulin bolus on BG levels in reaction to meal metabolism. The process commences with the examination of the BG information available for each sampled patient as a function of date and time (algorithm 1 in Textbox 1). Meal information in this study was used for annotation purposes (ie, to evaluate whether the BG peaks found were due to meals).

Textbox 1. **Peak detection algorithm (algorithm 1).**

Require: N ← BG files sorted by date;

 for i in len(N) do

  Apply low-pass filter to N_i_ to avoid noise;

  {P_1_, P_2_, P_3_, ..., P_i_} ← peaks;

  {W_1_, W_2_, W_3_, ..., W_i_} ← widths of peaks

  Obtain percentiles 95th of peak heights and widths;

  for j in len({P_1_, P_2_, P_3_, ..., P_i_}) do if P_j_ or W_j_ ≥ P95

   then

    T_ini_ ← T_Peak j_ − 1hr; ▷ Obtain initial times to remove.

    T _f in_ ← T_Peak j_ + 3hrs; ▷ Obtain final times to remove.

    if T _f in_ ≥ 23 : 59 : 59 then

     T _f in_ ← T _f in_ − 00 : 00 : 00; ▷ Obtain value to be removed next day.

     Read N_i+1_ file;

     CleanBGFile ← Cut BG values ∈ (00 : 00 : 00, T _f in_);

   end if

  end if

  CleanBGFile ← Cut BG values ∈ (T_ini_, T _f in_)

  end for

end for

**Figure 2.**
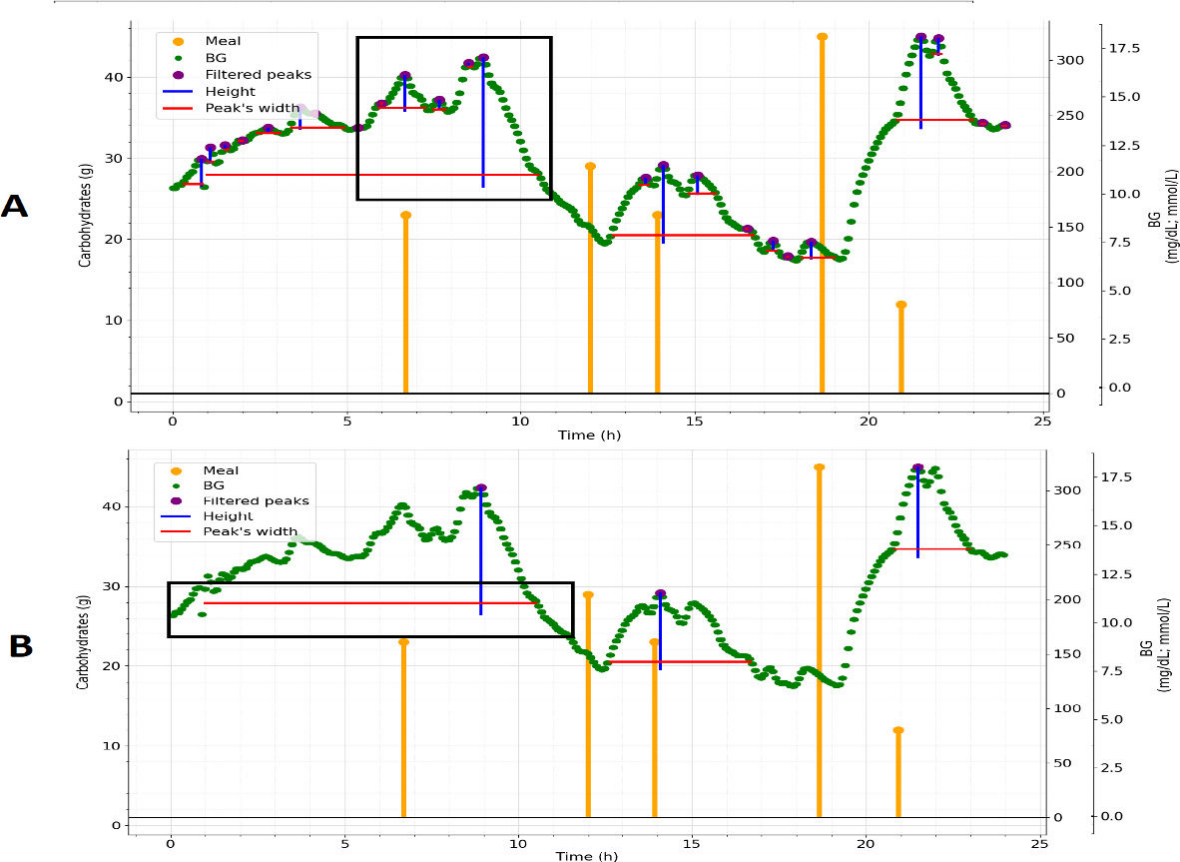
(A) This example illustrates a series of purple peaks identified by the algorithm over a single day. (B) After applying the outlier detector with threshold criteria based on the 95th percentile for width (hours) and height (mg/dL), only the peaks that meet these criteria are removed. In both examples, widths are shown in red, and heights are shown in blue. Meal information is highlighted in orange, offering visual guidance to help the reader better understand the phenomena evaluated by the algorithm. BG: blood glucose.

Algorithm 1 is specifically crafted to process BG data associated with a patient. In each iteration, the algorithm uses a low-pass filter to eliminate potential inaccuracies from reading errors in a CGM system. Once the filter is applied to the data, algorithm 1 takes on the task of detecting what is considered a BG peak for this study. This involves using algorithms for peak detection, height calculation, and width estimation, all based on standard signal processing techniques. The peak’s height (mg/dL) is calculated geometrically by measuring height relative to neighboring minima. Once the minima on each side of the peak are located, the height corresponding to the deeper valley is selected. The width (hours) is determined via linear interpolation at a specified height [22]. After identifying all peaks, an outlier detection method leveraging either height or width is used to pinpoint peaks potentially associated with meal intake. A threshold corresponding to the 95th percentile is established to filter out peaks likely linked to meals. The outcome of this process is illustrated in Figure 2, where panel A displays all detected peaks and panel B shows the filtered peaks following the application of the threshold. Similar approaches for outlier detection using peak characteristics such as height and width have been discussed in the literature, including robust regression and outlier detection methods [23], anomaly detection techniques [24], and event detection with outlier handling in biological time-series data [25]. Research shows that different macronutrients (carbohydrates, proteins, and fats) affect glucose peak profiles in distinct ways. Carbohydrates typically cause a rapid rise in BG, leading to a sharp increase followed by a quick, often asymmetrical decline. In contrast, proteins and fats slow gastric emptying and glucose absorption, resulting in a more delayed and flatter peak. These observations suggest that glucose peak curves are better modeled using skewed distributions such as log-normal or gamma distributions rather than the traditional symmetric Gaussian curve [26]. Furthermore, one study indicated that glucose use begins soon after a meal, with peak glucose use and insulin action typically occurring within 3 to 4 hours after a meal [27]. Therefore, for this study, if peaks were identified, a 4-hour window was excluded from the BG readings around each peak time (extending from 1 hour before to 3 hours after the peak).

**Figure 3.**
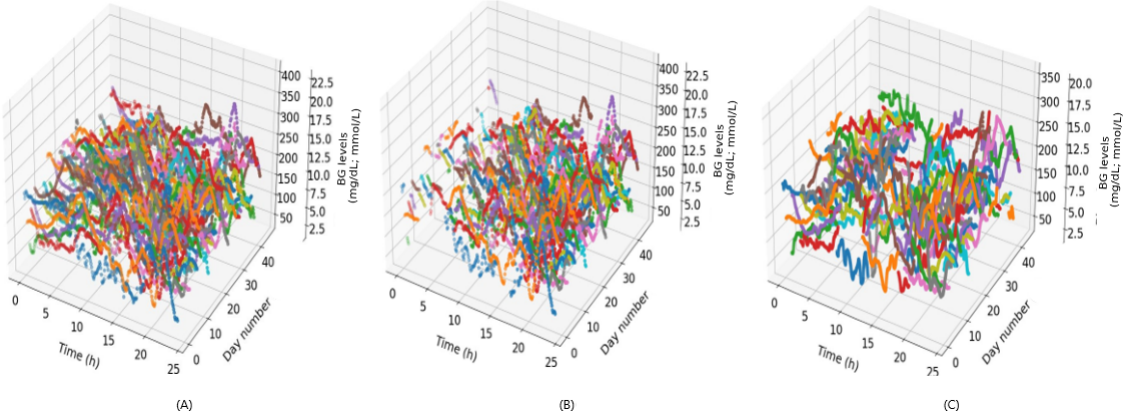
Example application of algorithm 1: (A) 45 blood glucose (BG) readings for patient ID 588 over a day; (B) segments of BG readings removed due to external influences; and (C) remaining BG levels after removal, representing periods without external interference.

If the temporal subtraction extends into the early hours of the subsequent day, this adjustment is carried over to the following reading. Consequently, the outcome is daily BG data in which any regions influenced by significant peaks in BG are effectively removed. It is essential to acknowledge that this step may be susceptible to inaccuracies as there could be instances in which peaks are not precisely obtained and some peaks may be attributed to factors other than meals. The effect of removing BG peaks using algorithm 1 is illustrated in Figure 3.

After removing BG intervals influenced by meal absorption, the algorithm consolidates the results into a unified table using a 24-hour pivot structure with 1-minute increments. This approach is selected to accommodate the irregular intervals at which measurements are recorded—typically every 5 minutes. The primary objective is to create a comprehensive daily composite, which serves as input for calculating BG relative change values.

**Figure 4.**
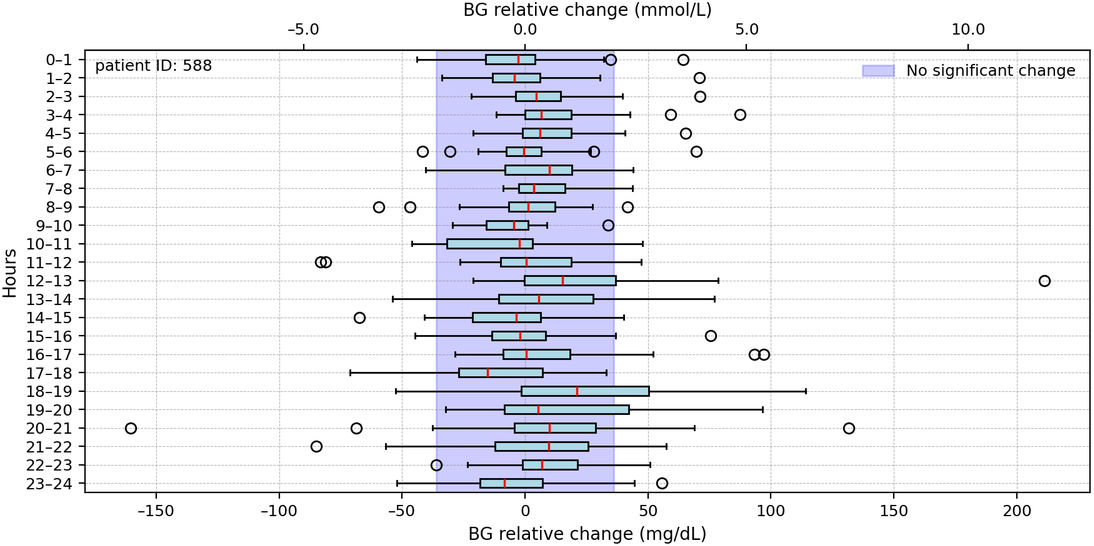
Hourly box plots depicting relative changes showcase the median distribution’s behavior over a composite 24-hour period. Values outside the blue-shaded range indicate greater fluctuations during those time slots; however, this range is intended solely for visualization purposes. BG: blood glucose.

Once the cleaned BG data are consolidated, they are divided into 24 one-hour segments for more detailed and thorough analysis. This segmentation aims to assess the stability of BG levels throughout the day. The underlying hypothesis is that, if the start and end points show no significant change, indicated by a relative BG change of 0 mg/dL (0 mmol/L), the BR is considered appropriate, and no further adjustments are needed. However, if differences are detected, further evaluation is required to determine the significance of these changes. The results of implementing this approach are shown in Figure 4.

The blue shading in Figure 4 represents a fixed range of –36 to +36 mg/dL (–2 to +2 mmol/L), serving as a visual reference for hourly fluctuations in relative BG levels. However, as the computation of box plots depends on the number of data points within each interval, some intervals exhibiting substantial variability require further analysis to assess the accuracy of these values.

To address this, a personalized reliability measure—based on the number of data points—was incorporated into the analysis. Percentiles were used as thresholds determined through exploratory analysis of the hourly data, with the 50th and 70th percentiles identified as points of notable separation in the dataset [28]. The resulting composite day representation illustrates relative changes in BG categorized using a 3-level reliability measure, as shown in the Peak Detection Analysis Results section, and is intended to evaluate the current deviation of the BR.

Finally, to assess whether this procedure serves as a viable alternative to the use of meal data, 2 tests were conducted. First, a noninferiority test was conducted to evaluate whether the cumulative deviation of BG relative changes captured by this method was comparable to that obtained using meal information. This metric was chosen because BG deviations are critical for assessing the current state of BR. Second, precision was calculated to measure the accuracy with which the method classified peaks as meal-related events. An interindividual analysis was also conducted to identify general patterns across participants.

### Noninferiority Test

**Figure 5.**
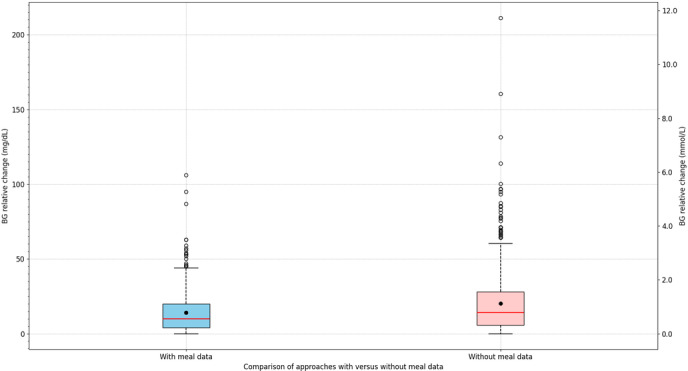
Box plot comparing the deviation in blood glucose (BG) between the method using meal data and the peak detection algorithm.

To determine whether the peak detection algorithm can be used as an alternative to traditional meal announcements, it was compared against historical meal data. Specifically, once a meal was announced, the subsequent 4 hours of BG data were excluded from the analysis, as recommended in prior studies [26,27]. The new method must demonstrate noninferiority by exhibiting comparable cumulative BG relative level deviations. As BG relative levels can fluctuate above and below the target value of 0 mg/dL, the absolute value of the deviation was used. A comparative distribution of BG deviations is shown in Figure 5.

### Precision

To further evaluate the method’s performance, precision was calculated to determine the proportion of detected peaks that were correctly attributed to meal events [29]. The analysis assessed whether the peak detection algorithm correctly identified a BG peak associated with a meal event occurring within a flexible window, from 1 hour before the estimated base starting point (defined as peak-width/2) to 1 hour after the peak. This window was defined based on exploratory data analysis, which revealed variability in the timing and quality of meal annotations (see the Precision Results section). The 95% CIs for these proportions were calculated using the Wilson score method.

### Interindividual Analysis

To identify general patterns across participants, the BG relative change data from all individuals were pooled into a single dataset, and each 24-hour composite day was divided into 1-hour intervals. For each interval, values were classified into 1 of 3 categories: excessive insulin (<0 mg/dL), insufficient insulin (>0 mg/dL), or optimal insulin (±0 mg/dL). For each category, the range (minimum and maximum), the average daily cumulative value, and the peak value across all participants were computed. Additionally, variance was compared between the excessive and insufficient insulin categories to assess consistency in glycemic deviations. The proportion of inappropriate BR settings was calculated as the number of hourly intervals classified as “excessive” or “insufficient” insulin divided by the total number of hourly intervals (n=288) after excluding periods influenced by meals.

## Results

### Overview

The results of the analysis are structured into 4 key stages of evaluation. First, the phenomena observed in all patients were identified and described, highlighting the detected patterns (see the Peak Detection Analysis Results section). Second, the noninferiority test was conducted for each individual to evaluate the safety of using this methodology compared with using meal data (see the Noninferiority Test Results section). Third, the precision of the method was assessed (see the Precision Results section). Finally, an interindividual analysis was conducted to evaluate the general state of BR across participants and whether they shared a common issue with their actual BR (see the Interindividual Analysis Results section). This 4-stage approach enabled a deeper understanding of the findings and their implications.

### Peak Detection Analysis Results

**Figure 6.**
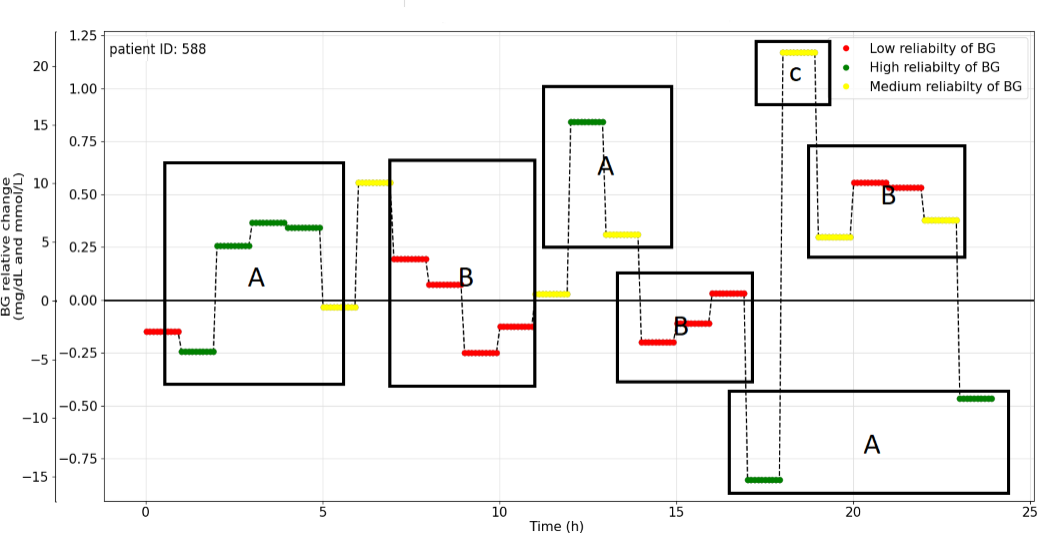
Blood glucose (BG) relative changes and reliability for the patient with ID 588. “A” indicates that BG levels demonstrated high reliability during early hours and periods between meals. “B” indicates that low-reliability BG values were observed around mealtimes. “C” indicates that occasional outliers were observed during periods of low reliability.

The analysis of BG relative levels across multiple patients revealed consistent patterns of reliability issues in the BG data. For most patients, higher reliability in BG relative changes was observed during early-morning hours, periods between meals, and late-night intervals. In contrast, mealtimes were associated with lower reliability, often accompanied by the presence of outliers. Patient ID 588 in Figure 6 serves as a representative example, where “A” shows BG levels with high reliability during early hours and between meals, “B” shows low-reliability BG values around mealtimes, and “C” highlights occasional outliers during periods of low reliability.

### Noninferiority Test Results

The results of the noninferiority test, which compared the level of deviation from optimal BG levels for each individual using this method and meal data, are shown in Table 2. There is no accepted standard for the noninferiority margin, but for this analysis, a threshold of 20% was established as a reasonable starting point. Using this margin, the majority of participants satisfied the noninferiority test. This indicates that the cumulative deviation of BG relative values as a metric was not worse when comparing this method to using meal information.

**Table 2.** Results of the noninferiority test for each participant in the OhioT1DM dataset.

ID	*t* test (*df*)	*P* value
540	3.381 (460)	<.001
544	–4.510 (980)	<.001
552	3.673 (1025)	<.001
567	4.344 (263)	<.001
584	6.955 (1336)	<.001
596	0.591 (854)	.277
559	0.663 (1101)	.254
563	3.345 (1299)	<.001
570	–2.219 (983)	.013
575	–7.310 (1080)	<.001
588	–3.192 (1140)	<.001
591	–1.396 (915)	.081

### Precision Results

The results, summarized in Table 3, indicate precision values ranging from 46.8% (88/188) of meals to 84% (158/188) of meals, with an average of 73.9% (139/188; SD 11.8%) of meals correctly classified per participant (95% CI 67.2%‐79.7%, calculated using the Wilson score method) when low-quality data, denoted by the “1” in the “low quality” column, were excluded. Notably, missing data (ie, instances in which no meal events were annotated in the dataset) impacted precision outcomes as the absence of reference points prevented meaningful comparisons.

**Table 3.** Summary of precision across multiple individuals. Low-quality data significantly impacted performance as the large proportion of missing information negatively affected the results^a^.

ID	Precision (%)	Low quality^b^	Missing meal files, n/N (%)
540	56	1	29/46 (63)
544	71	0	3/41 (7)
552	47	1	15/38 (39)
567	47	1	36/47 (77)
584	49	1	12/46 (26)
596	79	0	9/46 (20)
559	73	0	7/42 (17)
563	63	0	4/46 (9)
570	73	0	6/41 (15)
575	81	0	4/46 (9)
588	84	0	1/46 (2)
591	70	0	6/46 (13)

a Precision values ranged from 47% (88/188) to 84% (158/188) of meals, with an average of 74% (139/188) of meals in the filtered dataset.

b Low quality: binary indicator; 1=low-quality data, 0=high-quality data.

### Interindividual Analysis Results

**Figure 7.**
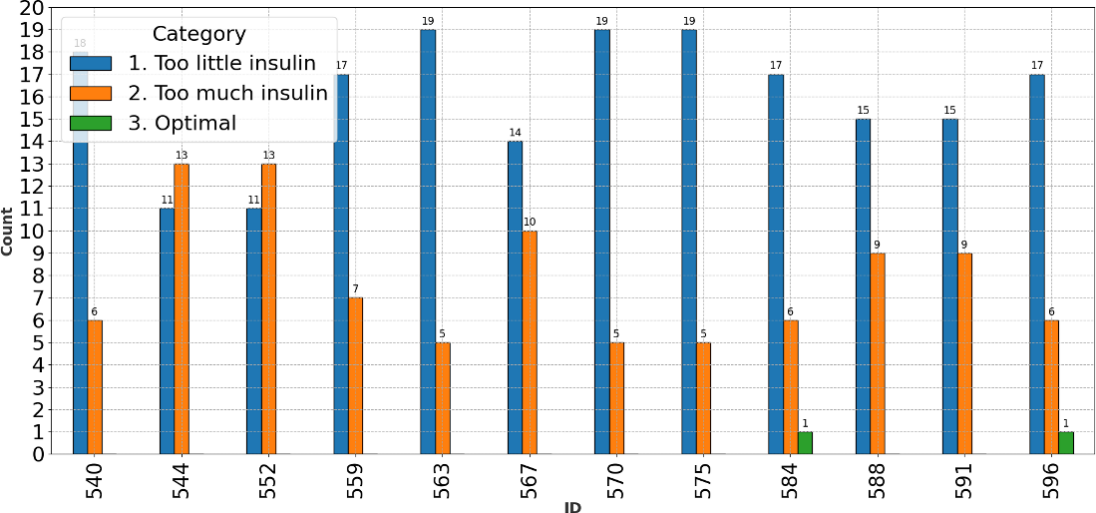
Histogram illustrating the distribution of hours during which individuals experienced insufficient, excessive, or optimal insulin levels, corresponding to blood glucose relative values that were below or above the optimal target or at the optimal target, respectively. Across 12 participants (24 hours each; total=288 values), 99.3% (286/288; SD 0.5%) of the values fell into the categories of either insufficient or excessive insulin. Optimal insulin levels were rare, averaging approximately 0.166 hours per day across the study group.

Figure 7 shows that 99.3% (286/288) of the BG relative values (SD 0.5%; 95% CI 97.5%‐99.8%) fell into the categories of either insufficient or excessive insulin, presenting a stark contrast to the relatively sparse occurrences of optimal insulin levels, averaging approximately 0.166 hours per day across the study group.

**Figure 8.**
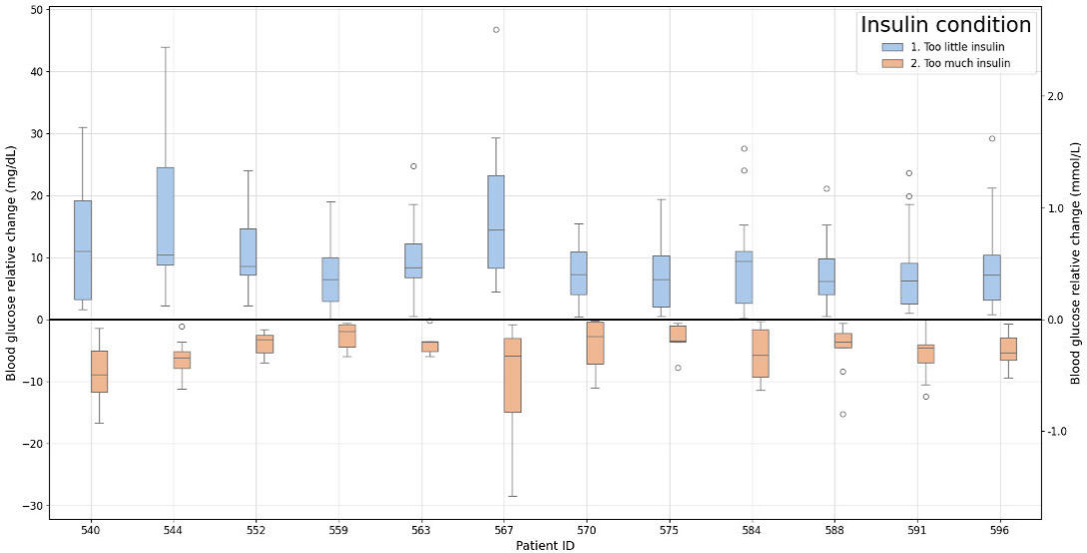
Box plots showing blood glucose relative levels under conditions of insufficient and excessive insulin administration, with interindividual variability ranging from −28.5 to 46.7 mg/dL (−1.58 to 2.59 mmol/L). Daily cumulative averages across all individuals revealed a mean of 200.6 (SD 61.7) mg/dL (11.1, SD 3.4 mmol/L; 95% CI 161.4–239.8 mg/dL), peaking at 270.15 (SD 14.99) mmol/L.

Figure 8 shows that BG relative levels seemed to exhibit diverse patterns when either too little or too much insulin was administered, with noticeable variations among individuals, ranging from −28.5 to 46.7 mg/dL (−1.58 to 2.59 mmol/L). Data analysis indicates that, on average, individuals show lower variability when more insulin than optimal is administered compared to other dosing conditions. When computing the daily cumulative average from the data of all individuals, the analysis revealed an average value of 200.6 mg/dL (SD 61.7; 11.1 mmol/L, SD 3.4; 95% CI 161.4–239.8 mg/dL), with peak values reaching 270.15 (SD 14.99) mmol/L.

## Discussion

### Principal Results

This study introduced a meal-independent approach for assessing the adequacy of BR using historical CGM and insulin delivery data. Unlike prior work focused on BR as an input for BG prediction models, our method directly evaluates BR performance over time and identifies when adjustments are needed.

Peak detection analysis across participants revealed consistent patterns in data reliability—higher reliability was generally observed during early-morning hours, between meals, and late at night, whereas mealtimes tended to show lower reliability and more frequent outliers.

Using a predefined 20% noninferiority margin on absolute BG relative change, the no-meal method showed comparable behavior to the meal-based approach in 9 of 12 participants (1-sided *P*<.05). These findings indicate that the method may achieve comparable performance without relying on meal information, although individual differences and data variability may explain the few nonsignificant results. Precision averaged 73.9% (139/188) of meals correctly classified per participant (SD 11.8%; 95% CI 67.2%‐79.7%), indicating reasonable accuracy in excluding meal-related BG excursions.

Interindividual analysis showed that 99.3% (286/288) of BG relative values (SD 0.5%; 95% CI 97.5%‐99.8%) fell into the excessive or insufficient insulin categories, with deviations ranging from −25.5 to 46 mg/dL (−1.58 to 2.59 mmol/L). These findings highlight the prevalence of suboptimal BR settings and suggest that the proposed approach could support BR adjustments without the burden of collecting meal data, reducing patient effort and device-related strain [30] and supporting future calibration in both closed-loop and stand-alone pump use.

Although CGM-derived glucose trends formed the primary analytical focus, basal insulin delivery data were central to interpreting these results. The composite 24-hour BG profile for each participant was generated directly from their CGM data, and relative BG changes were assessed in the context of BR. Given that 99.3% (286/288; SD 0.55%) of the BG relative values fell into the excessive or insufficient categories rather than the optimal 0 mg/dL range, these deviations strongly indicate that the current BR configurations were inadequate. In this way, insulin delivery data were not only included but also essential for linking glucose deviations to inaccuracies in BR setup.

### Comparison With Prior Work

Extensive research in BG prediction for T1DM has been conducted in recent years [31]. Approaches have ranged from data-driven models to physiological models and combinations of both, often using advanced machine learning techniques, related algorithms, or mathematically complex differential equations [32,33]. This work contributes to that body of knowledge by introducing an algorithm that may help assess whether current BR settings are appropriate without requiring meal data. This is clinically relevant because incorrect BR inputs—whether in closed-loop systems that revert to manual mode or in stand-alone insulin pumps—can lead to inaccurate adjustments and suboptimal BG management.

Previous research efforts have predominantly revolved around BR as an input variable rather than it being the central subject of investigation. Few studies have tackled this challenge directly. Certain works have applied clustering methods, grouping new patients based on characteristics such as age or diabetes duration [34]. Several studies have used traditional machine learning methods for prediction tasks [35]. However, test outcomes have been suboptimal [36]. Recent research [37,38] has documented the effective performance of automatic insulin delivery systems in real-world operation. While they offer significant clinical benefits, these systems still have calibration challenges and emerging cybersecurity considerations [5].

In addition to the previous examples, some studies have examined the characterization of BR profiles. Such studies have examined the previously noted variables [10,39-41] alongside factors including the dawn phenomenon and age-related effects on these profiles. However, these studies do not provide guidance on how to accurately determine the appropriate BR for an individual, nor do they address how these rates may change over time for the same person.

In the landscape of meal detection research, the consulted examples hinge on the computation of rates of change using sophisticated mathematical approaches such as Kalman filters [42-45] or the validation of probabilistic methods through testing in in silico environments [46-48].

### Limitations

This work acknowledges several limitations. First, it relies on the assumption that mealtimes align with the attributes defined in the methodology—specifically, that BG peaks exceed the 95th percentile for either width (hours) or height (mg/dL). Second, the dataset was constrained by a limited sample size (N=12) and its demographic characteristics and data quality, which inherently impacts the precision and generalizability of the analysis. Third, the algorithm has only been evaluated on the dataset used for its development; therefore, validation in independent external datasets will be necessary to establish robustness and applicability across broader populations. Finally, while this study did not assess the clinical significance of the observed variations and changes in BG levels, this will be comprehensively addressed in future clinical investigations.

### Conclusions

This paper highlights the preliminary potential of the proposed algorithm, which relies solely on CGM data to approximate the timing of meal-related glucose excursions. By excluding these instances, the algorithm may help provide a clearer assessment of relative BG deviations and the current state of BR. These findings should be interpreted cautiously as preliminary evidence, and validation in larger and independent cohorts will be required before any clinical application can be considered. Therefore, future work will focus on refining the method and assessing its utility for supporting BR adjustments aimed at reducing BG deviations and extending TIR, particularly in LMICs, where advanced systems are less common.

## Abbreviations

BG: blood glucose
BR: basal rate
CGM: continuous glucose monitoring
LMICs: low- and middle-income countries
PBT: personalized basal tuner
T1DM: type 1 diabetes mellitus
TIR: time in range
